# Oxidative Stress Response of *Aspergillus oryzae* Induced by Hydrogen Peroxide and Menadione Sodium Bisulfite

**DOI:** 10.3390/microorganisms7080225

**Published:** 2019-07-30

**Authors:** Huanhuan Shao, Yayi Tu, Yijing Wang, Chunmiao Jiang, Long Ma, Zhihong Hu, Jiangfan Wang, Bin Zeng, Bin He

**Affiliations:** 1 Jiangxi Key Laboratory of Bioprocess Engineering and Co-Innovation Center for In-vitro Diagnostic Reagents and Devices of Jiangxi Province, College of Life Sciences, Jiangxi Science & Technology Normal University, Nanchang 330013, China; 2 College of Life Sciences, Sichuan Normal University, Chengdu 610101, China

**Keywords:** *Aspergillus oryzae*, oxidative stress, antioxidant enzymes, glutathione, fatty acids

## Abstract

Oxidative stress response protects organisms from deleterious effects of reactive oxygen species (ROS), which can damage cellular components and cause disturbance of the cellular homeostasis. Although the defensive biochemical mechanisms have been extensively studied in yeast and other filamentous fungi, little information is available about *Aspergillus oryzae*. We investigated the effect of two oxidant agents (menadione sodium bisulfite, MSB, and hydrogen peroxide, H_2_O_2_) on cellular growth and antioxidant enzyme induction in *A. oryzae*. Results indicated severe inhibition of biomass and conidia production when high concentration of oxidants was used. Transcriptomic analysis showed an up-regulated expression of genes involved in oxidoreduction, such as catalase, glutathione peroxidase, and superoxide dismutase. In addition, it was observed that oxidative stress stimuli enhanced the expression of Yap1 and Skn7 transcription factors. Further, metabolomic analysis showed that glutathione content was increased in the oxidative treatments when compared with the control. Moreover, the content of unsaturated fatty acid decreased with oxidative treatment accompanying with the down-regulated expression of genes involved in linoleic acid biosynthesis. This study provided a global transcriptome characterization of oxidative stress response in *A. oryzae*, and can offer multiple target genes for oxidative tolerance improvement via genetic engineering.

## 1. Introduction

*Aspergillus oryzae* (*A. oryzae*) has been used for the production of traditional fermentation such as soy sauce, miso, and douchi, and demonstrated promising potential for primary and secondary metabolites [1,2]. In additional, *A. oryzae* has been listed as “Generally Recognized as Safe (GRAS)” by the Food and Drug Administration (FDA), and its safety has been recognized by the World Health Organization (WHO) [3]. Like other microorganisms, *A. oryzae* has been subjected to pressure from internal and external environment during the process of growth and fermentation. Under normal aerobic condition, the metabolic byproduct of *A. oryzae* may result in the formation of reactive oxygen species (ROS), mainly including superoxide anion, hydroxyl radical, and hydrogen peroxide. The accumulation of ROS can damage the cellular components due to their stronger reactivity than molecular oxygen [4]. As a consequence, ROS can cause disturbance of cellular homeostasis, which may lead to the inactivation of enzymes and disruption of unsaturated fatty acid double bond, even resulting in cell ageing or death [5]. Microorganisms have evolved several defense mechanisms against the deleterious effects of ROS. The most detailed studies on the response to oxidative stress have been reported in *Saccharomyces cerevisiae* [6]. However, the biochemical defense mechanisms of *A. oryzae* against oxidative stress have not been completely elucidated, which is essential for the survival and adaptation to an oxidative environment in fermentation and production process.

In *S. cerevisiae*, there are two intricate antioxidant defense systems: enzyme defense and non-enzyme defense system [6]. The antioxidant enzymes, mainly including catalase (CAT), glutathione peroxidase (GPX) and superoxide dismutase (SOD), played an important role in the defense against ROS by catalytically reduce it through electron transfer. There are two SOD isoenzymes in *S. cerevisiae* (SOD1, SOD2), which catalyze the two-step disproportionation of superoxide anion to hydrogen peroxide and oxygen molecule. Deletion of SOD genes caused an increased sensitivity to oxidative stress-inducing agents, or decreased the level of reduced glutathione (GSH) [7]. Moreover, the Δsod mutant exhibited oxidative stress-related variations in cells, low growth rate in air, and inability to grow in an atmosphere of 100% oxygen in a rich cultivation medium [8]. Furthermore, the loss of SOD dramatically reduced the chronological and replicative lifespans of the yeast [9]. CAT is an iron-containing enzyme, which catalyzes the disproportionation of H_2_O_2_ into O_2_ and H_2_O molecules. In yeast, there are two CAT isoenzymes, including CTT1 located in the cytosol and CTA1 located in peroxisomes and mitochondria [10]. However, the loss of either individual catalase or both CTT1 and CTA1 exhibited no effect on H_2_O_2_ sensitivity [11]. The results indicate that there are other peroxidases, such as GPX, that can effectively remove H_2_O_2_ in yeast cell. GPX can catalyze the reduction of H_2_O_2_ or phospholipid hydroperoxides to H_2_O or corresponding alcohol using reduced glutathione [12]. The biological purpose of oxidative stress response in the first place is to reinstate the cellular redox homeostasis. This requires the regulation of transcriptional changes via Yap1 and Skn7 transcription factors responsible for the expression of key genes coding enzymes that are required for ROS detoxification [13]. Yap1 is a major regulator of oxidative stress response in *S. cerevisiae*, which is distributed mainly in the cell cytosol [14]. Skn7 is a constitutive nuclear protein that usually functions in combination with Yap1 in the response to peroxides. Approximately half of the genes controlled by Yap1 are regulated by both Yap1 and Skn7 (including SOD1 and SOD2), while the other half is regulated in a Skn7-independent manner [15,16].

The non-enzyme defense system reinstates the cellular redox homeostasis through the modifications of cellular components, such as glutathione and lipid. Glutathione plays an important role in the response to oxidative stress that might be the target of oxidation against the exposure to an exogenous oxidant. Glutathione can protect the mitochondria from oxidant damage by reacting with oxidative agent [17,18]. When the level of ROS exceeds the antioxidant capacity of cells, lipids can react with ROS to reinstate the cellular redox homeostasis as well. Under oxidative stress, oxidants attack lipids containing C-C bond(s), particularly polyunsaturated fatty acids [19,20]. Since yeast does not contain polyunsaturated fatty acids, there is no lipid peroxidation process in its cells. Thus, it is imperative to study the biochemical defense mechanisms against oxidative stress in filamentous fungi, such as *A. oryzae*. Few filamentous fungi, such as *Neurospora crassa*, *Aspergillus niger*, *Aspergillus fumigatus,* and *Aspergillus nidulans*, have been investigated the defense mechanisms against oxidative stress [21,22,23,24]. Although it is an important industrial microorganism for the production of traditional fermentation and enzymes, there is a scarcity of information about the responses of *A. oryzae* against various oxidative stresses. The objective of this study was to understand the response of *A. oryzae* to oxidative stress. Thus, two types of ROS generating agents were examined for their effects on cellular growth and antioxidant enzyme induction in *A. oryzae*.

## 2. Materials and Methods

### 2.1. Effect of Oxidative Stress on Aspergillus oryzae Growth

*Aspergillus oryzae* (*A. oryzae*) 3.042 (China center of industrial culture collection (CICC) no. 40092), identified with morphological characteristics and ITS gene sequence, was selected to examine the response to oxidative stress. For inoculum preparation, cryopreserved conidia were activated in fresh potato dextrose agar (PDA) medium and incubated at 30 °C for 3 days. Conidia were then harvested and suspended in sterile water for quantitative determination of conidia concentration using a hemocytometer [25]. Oxidative stress treatments were performed on solid PDA medium supplemented with stress-initiating agents hydrogen peroxide (H_2_O_2_ at final concentration of 3 mM and 6 mM) and menadione sodium bisulfite (MSB at final concentration of 0.28 mM and 0.42 mM) [26]. PDA medium absent of oxidant was used as control. Freshly prepared suspension (1 × 10^7^ conidia) was inoculated per plate covered by cellophane, and then all cultures were placed at 30 °C for 72 h in darkness. Then, fungal mycelia were dried overnight at 65 °C for the biomass determination and the density of spores was determined by hemocytometer. For the determination of reduced glutathione and fatty acids, samples were collected in the same way. Three replicates were used for each sample in above experiments and images of the most representative colonies were taken.

### 2.2. Determination of Reduced Glutathione Levels

For the determination of reduced glutathione (GSH), a previously described method (5,5’-dithiobis-(2-nitrobenzoic acid) (DTNB) method) was used [27]. In brief, cells were harvested and washed twice with 1 mM phosphate buffered saline (pH 7.4) to remove any traces of growth medium. Cells were dried overnight at 65 °C. Dry mycelia (100 mg) were mixed with GSH assay buffer and grind with liquid nitrogen. The grinded cells were centrifuged at 8000 g for 10 min at 4 °C and the suspension obtained was used to determine GSH levels. GSH quantification was performed by incubating the reaction mixture containing crude extract and 3 mM of DTNB at 30 °C for 5 min followed by NADPH addition (0.4 mM) and 2 μL of glutathione reductase (GR) enzyme. Samples were kept for another 2 min to allow the reaction completed and absorbance was measured at 412 nm. GSH levels in the samples were compared with the standard curve prepared by using various concentrations (50, 100, 150, 200 μg/mL) of GSH.

### 2.3. Measurement of Intracellular Fatty Acid Content

Total lipids were extracted and quantified following a previously described method [25]. Cells were harvested and incubated in chloroform with 2% H_2_SO_4_-MeOH solution at 70 °C for 2 h to obtain fatty acid methyl esters (FAMEs). FAMEs components were separated and analyzed using a coupled QP2010 gas chromatography–mass spectrometry (GC-MS) (Shimadzu, Kyoto, Japan). The system was equipped with Supelco SP-2340 fused silica capillary column (30 m × 0.25 mm i.d., film thickness of 0.2 μm; Bellefonte, PA, USA). FAMEs were identified by comparing their mass spectra with a spectrum database. Fatty acid peaks were identified by comparing retention times with external standards or similarity search. Fatty acid quantification was performed by using the peak area of the most intensive ion of each peak [25,28].

### 2.4. RNA Extraction and Illumina Sequencing

Total RNA was extracted from all samples using Trizol reagent (Roche, Basel, Switzerland) following the manufacturer’s instructions and genomic DNAs were digested by DNase I (Fermentas, Waltham, MA, USA). RNA concentration was analyzed by Qubit fluorometer (Invitrogen, Carlsbad, CA, USA) and its quality was determined using Agilent 2100 Bioanalyzer. Further, mRNA was enriched from the total RNA using Oligo (dT) magnetic beads and fragmented into about 200 nt using fragmentation buffer. These fragments were then reverse transcripted into cDNA with random primers and the second-strand cDNA was synthesized using DNA polymerase I and RNase H. The resulting double-stranded cDNA was purified, end repaired and ligated to Illumina sequencing adapters. The cDNA sequencing was performed using Illumina HiSeq 2500 (Biomarker Biotechnology Co., Beijing, China).

### 2.5. Differentially Expressed Genes Analysis

After the removal of low-quality reads, adaptor sequences and reads with N (unknown nucleotides) exceeding 10%, clean-read datasets were obtained and aligned to *A. oryzae* 3.042 reference genome using Tophats2 (v2.0.3.12) [29]. Gene abundances were quantified by RSEM software and the quantification of gene expression level was normalized using FPKM (Fragments Per Kilobase of transcript per Million mapped reads) method [30]. Differentially expressed genes (DEGs) in all samples were identified using DESeq R package [31]. The log2 (fold change) over 1 and false discovery rate (FDR) within 0.05 were set as the threshold for significant DEGs [25]. DEGs were used for the enrichment analysis of GO functions and KEGG pathways.

### 2.6. Calculations and Statistical Analyses

Three independent experiments were performed and the values shown in this study were presented as mean ± standard error (SE). The effects of treatments were compared by one-way nested analysis of variance (ANOVA), followed by least significant difference test (LSD) for mean comparison. All statistical analysis was performed using SAS 9.20 software (SAS Institute Inc., Cary, NC, USA) at *p* < 0.05.

## 3. Results

### 3.1. Effect of Oxidant Species and Concentration on A. oryzae Cell Growth

**Figure 1:**
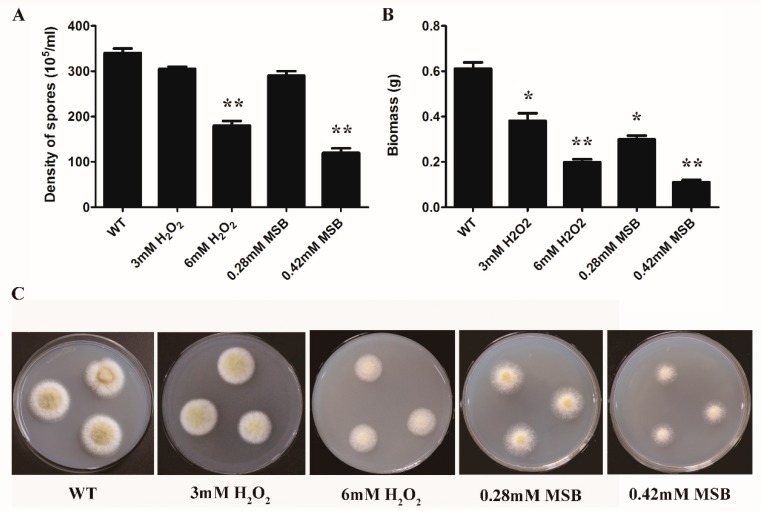
The density of spores, dry biomass and phenotype as affected by oxidative treatment. (**A**) The density of spores in the oxidative treatments (H_2_O_2_ and MSB) and the control; (**B**) The dry biomass in the oxidative treatments and the control. The mycelia were collected by peeling them off from the plates and dried overnight for the determination of biomass; The bars represent the average (±SE) of biological repeats. (**C**) The phenotype of *A. oryzae* in the control and the oxidative treatment at 72 h. The colony growth was retarded and the conidia production was inhibited. WT: the control; H_2_O_2_: the H_2_O_2_ treatments (3 and 6 mM); MSB: the MSB treatments (0.28 and 0.42 mM).

To study the effect of oxidant species and its concentration on *A. oryzae* growth and development, the density of spores, dry biomass, and phenotype analysis were determined and performed. When *A. oryzae* cells were supplemented with 3 mM H_2_O_2_ and 0.28 mM MSB, no significant effects were observed on the spore density between control and oxidative treatments, while the dry biomass was decreased when compared to control (Figure 1A,B). Under high H_2_O_2_ (6 mM) and MSB (0.42 mM) concentration, the density of spores and dry biomass of *A. oryzae* showed a significant difference between the oxidative treatments and control. During the cultivation period, the cell growth and conidia formation was notably inhibited with high concentration of oxidant (6 mM H_2_O_2_ and 0.42 mM MSB) compared to others, indicating severe inhibition of biomass and conidia production (Figure 1C).

### 3.2. Global Analysis of A. oryzae Transcriptome under Oxidative Stress

To investigate the oxidative stress response of *A. oryzae* induced by H_2_O_2_ and MSB, a transcriptome analysis based on RNA sequencing of *A. oryzae* that was subjected to various oxidative stress treatments (no oxidant, 3 mM and 6 mM H_2_O_2_, 0.28 mM and 0.42 mM MSB) was performed. This resulted into the generation of 40.79, 41.41, 41.10, 43.61 and 39.90 million clean reads per library, respectively. The Q30 values (99.9% accuracy of bases) were higher than 94% for all samples, indicating the good quality of sequencing data. The clean reads were aligned to *A. oryzae* 3042 genome sequence, and more than 84% of the clean reads for each sample were uniquely mapped to the genome (Table 1).

### 3.3. Differentially Expressed genes Analysis of A. oryzae Transcriptome under Oxidative Stress

**Figure 2:**
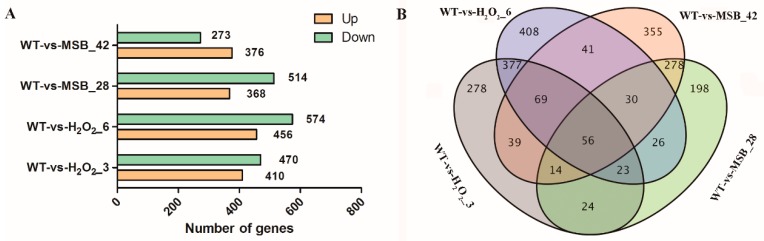
Distribution of differentially expressed genes (DEGs) in the five samples. (**A**) DEGs’ distribution between control and oxidative treatment; (**B**) Venn diagram exhibiting the DEGs’ distribution in five samples. WT: the control; H_2_O_2_: the H_2_O_2_ treatments (3 and 6 mM); MSB: the MSB treatments (0.28 and 0.42 mM).

The relative expression level of each gene in terms of FPKM was estimated by mapping all clean reads from each library back to the reference genome. Compared to the control, a total of 880 genes were identified as DEGs at 3 mM H_2_O_2_, which comprised 410 up-regulated genes (accounting for 47% of all significant DEGs) and 470 down-regulated genes (accounting for 53%). Between control and 6 mM H_2_O_2_ treatment, 1030 DEGs were identified, including 456 up-regulated genes (44%) and 574 down-regulated genes (56%). Under 0.28 and 0.42 mM MSB treatments, 882 and 649 DEGs were identified (Figure 2A). A Venn diagram of DEGs distribution was constructed (Figure 2B), and it was observed that only 56 DEGs were commonly shared among the four groups. The numbers of specific DEGs between 6 mM H_2_O_2_ treatments and control (408) as well as 0.42 mM MSB treatments and control (355) were remarkably greater than that of the other two groups (Figure 2B). This suggested the involvement of complex developmental events at high concentration of oxidant treatments (Figure 2B). In addition, we found that more common DEGs were found between WT-vs-H_2_O_2__3 and WT-vs-H_2_O_2__6 or WT-vs-MSB_28 and WT-vs-MSB_42 when compared to that between WT-vs-H_2_O_2_ and WT-vs-MSB, suggesting that the response of *A. oryzae* to H_2_O_2_ and MSB might be different.

### 3.4. Expression Analysis of Genes Involved in Oxidoreduction under Oxidative Stress

**Figure 3:**
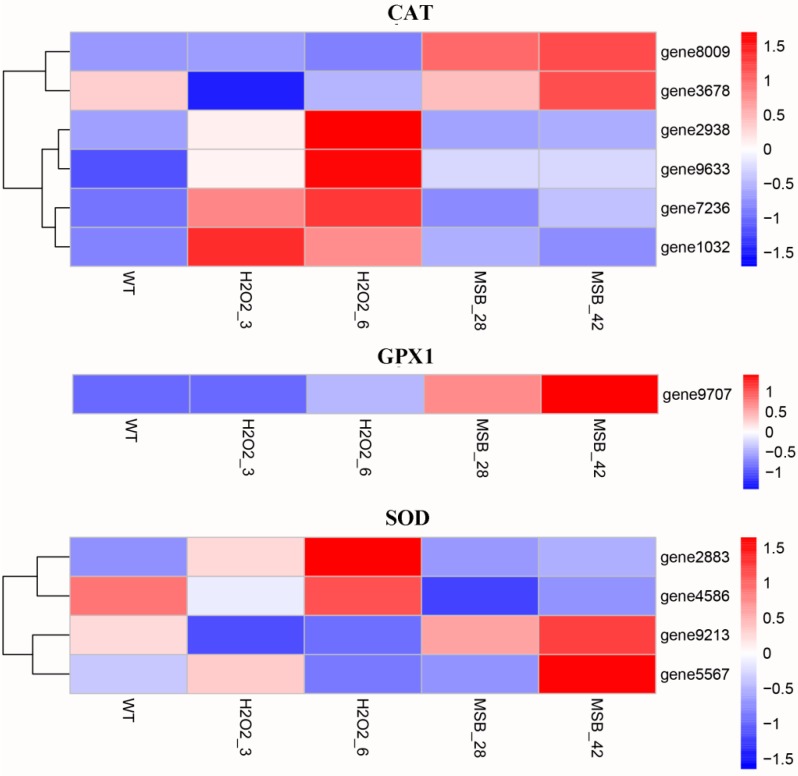
The expression profile of catalase (CAT), GPX1 (glutathione peroxidase) and superoxide dismutase (SOD) in the five samples. WT: the control; H_2_O_2_: the H_2_O_2_ treatments (3 and 6 mM); MSB: the MSB treatments (0.28 and 0.42 mM).

Under oxidative stress, fungus could eliminate the effects of reactive oxygen species (ROS) reactions through the expression genes involved in oxidoreduction, including CAT, GPX, and SOD [32]. Therefore, the genes encoding CAT, GPX and SOD were identified in *A. oryzae* transcriptome and their expression profile was analyzed. As shown in Figure 3, six CAT genes, including gene8009, gene3678, gene2938, gene9633, gene7236, and gene1032 were identified in *A. oryzae*. The results of expression analysis showed that only two CAT genes (gene8009 and gene3678) were up-regulated in MSB treatments, while other four CAT genes (gene2938, gene9633, gene7236, and gene1032) were significantly up-regulated in H_2_O_2_ treatments. Furthermore, only one GPX gene was identified in *A. oryzae* and notably up-regulated at MSB treatments. The four SOD genes showed a different expression profile under H_2_O_2_ and MSB treatments. Two SOD genes (gene2883 and gene4586) exhibited a higher expression level in samples cultivated in solid PDA medium supplemented with H_2_O_2_, while the remaining two SOD genes (gene9213 and gene5567) exhibited highest expression level under MSB treatments. The different expression patterns of 11 genes involved in oxidoreduction under H_2_O_2_ and MSB treatments suggested that the mechanism of *A. oryzae* in response to various oxidants is complex and diverse.

### 3.5. Expression Analysis of Transcription Factors Possibly Involved in Oxidative Stress Response

**Figure 4:**
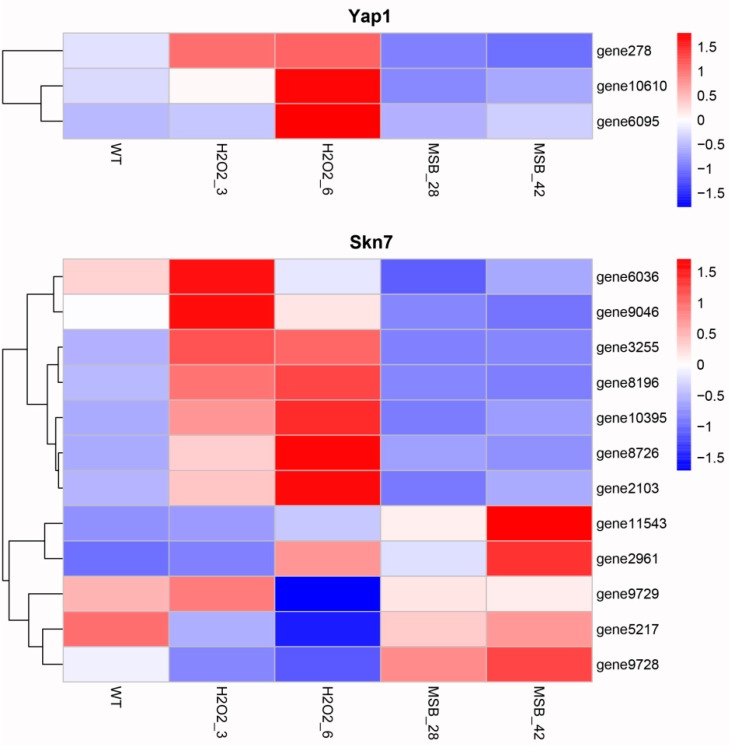
The expression profile of Yap1 and Skn7 transcription factors in the five samples. WT: the control; H_2_O_2_: the H_2_O_2_ treatments (3 and 6 mM); MSB: the MSB treatments (0.28 and 0.42 mM).

Previous studies have reported that several transcription factors, such as Yap1 and Skn7, responded to oxidative stress through maintaining thiol redox homeostasis [15]. Thus, we identified and analyzed the expression profile of Yap1 and Skn7 transcription factors. A total of three Yap1 transcription factors (gene278, gene10610, and gene6095) were identified in *A. oryzae*, and they were highly expressed in the samples cultivated in solid PDA medium supplemented with H_2_O_2_ (Figure 4). Similarly, 12 Skn7 transcription factors were identified in *A. oryzae* transcriptome. Seven Skn7 transcription factors exhibited significant increase in the expression levels when treated with H_2_O_2_, while the remaining five Skn7 transcription factors showed up-regulated expressions in MSB treatments. These results suggested that the response of *A. oryzae* to H_2_O_2_ and MSB treatment might be different.

### 3.6. Effects of Oxidant on A. oryzae GSH

**Figure 5:**
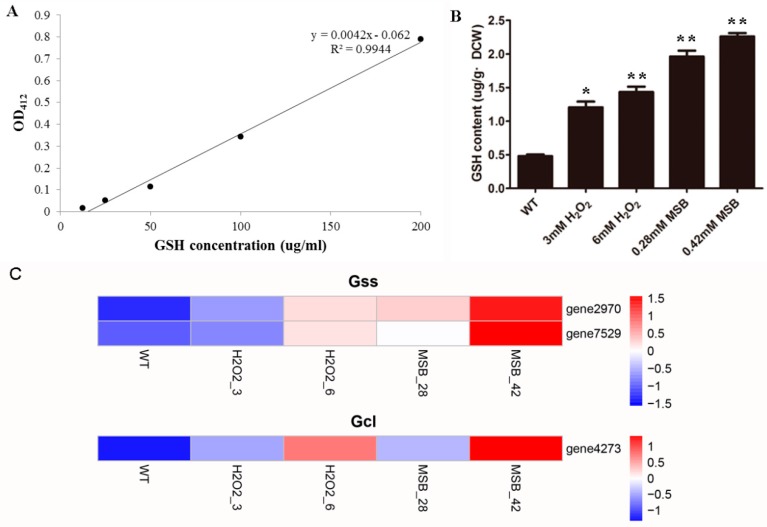
The content of glutathione (GSH) and expression profile of genes involved in glutathione biosynthesis in the oxidative treatments (H_2_O_2_ and MSB) and the control. (**A**) The standard curve of GSH concentration. (**B**) The changes of GSH level in response to oxidative stress. The bars represent the average (±SE) of biological repeats. The content of GSH increased with the increasing concentration of H_2_O_2_ and MSB. (**C**) The expression pattern of DEGs involved in the linoleic acid biosynthesis pathways (from left to right: the control, 3 mM and 6 mM H_2_O_2_, 0.28 mM and 0.42 mM MSB).

GSH plays an important role in the response to oxidative stress as the most important antioxidant through the enzymes involved in oxidoreduction and Yap1 and Skn7 transcription factors. We determined the content of *A. oryzae* GSH in the oxidative treatments through DTNB method. As we expected, the content of GSH was found to be increased in oxidative treatments compared to control (Figure 5). In addition, GSH content under MSB stress was higher than those under H_2_O_2_ stress. To further confirm the increase of glutathione level, the expression profile of genes involved in glutathione biosynthesis, including glutamate-cysteine ligase (Gcl) and glutathione synthase (Gss), was investigated. The results revealed that two genes encoding Gss and the gene encoding Gcl both exhibited highest expression levels in 0.42 mM MSB treatments, which were supported the increase of GSH content.

### 3.7. Change in Linoleic acid Biosynthesis under Oxidative Stress

**Figure 6:**
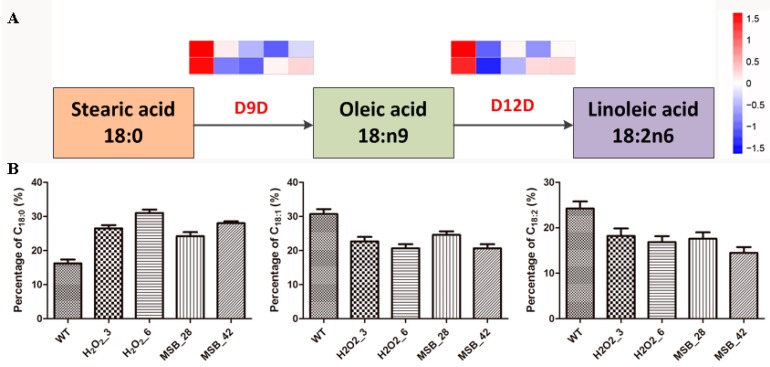
DEGs involved in the linoleic acid biosynthesis pathways and the changes of C18 fatty acid in response to oxidative stress. (**A**) The expression pattern of DEGs involved in the linoleic acid biosynthesis pathways (from left to right: the control, 3 mM and 6 mM H_2_O_2_, 0.28 mM and 0.42 mM MSB); (**B**) The changes of C18 fatty acid in response to oxidative stress. The bars represent the average (±SE) of biological repeats. WT: the control; H_2_O_2_: the H_2_O_2_ treatments (3 and 6 mM); MSB: the MSB treatments (0.28 and 0.42 mM).

According to KEGG pathway analysis, four genes involved in *A. oryzae* linoleic acid biosynthesis were identified. They encoded delta 9 fatty acid desaturases (D9D) and delta 12 fatty acid desaturases (D12D). D9D and D12D were rate limiting enzymes in the biosynthesis of unsaturated fatty acid and polyunsaturated fatty acids, respectively. Analysis of gene expression profiling for D9D and D12D revealed that the four genes involved in linoleic acid biosynthesis were down-regulated in the response to oxidative stress, suggesting that unsaturated fatty acid synthesis was inhibited in *A. oryzae* cells in response to oxidative stress (Figure 6A). This was further supported by the determination of fatty acid content, showing a decrease in unsaturated fatty acid (Figure 6B).

## 4. Discussion

Environmental stress responses of *A. oryzae* cells are not isolated in linear metabolic or signaling pathways. Global responses have been associated with a much larger stress signaling network that integrates the information from various pathways and enzymes [6]. Therefore, a systematic analysis method is required to observe the intracellular global regulation patterns. Genome-wide approaches are considered as a convenient and efficient method to characterize gene expression profiles and elucidate the whole regulation network in plants, animals and microorganisms. The genome-wide projects of *A. oryzae* involved a lot of effort, and the complete genome sequence of *A. oryzae* was obtained through whole-genome shotgun approach in 2005 [2,33]. Wang et al. analyzed the transcriptomes of *A. oryzae* grown on solid-state culture, and in liquid culture with and without DTT treatment to understand the complex mechanism in *A. oryzae* at whole genome level [34]. A comprehensive transcriptome analysis of *A. oryzae* was performed to understand the possible biological processes across various growth stages [35]. In this study, genome-wide response of gene expressions in *A. oryzae* under oxidative conditions was analyzed to better understand the oxidative stress response of *A. oryzae* induced by H_2_O_2_ and MSB.

The results of this study showed that exposure of *A. oryzae* cells to H_2_O_2_ and MSB led to a substantial transcriptional regulation. However, the response of *A. oryzae* cells to H_2_O_2_ and MSB treatment was different. Only 56 DEGs were found to be commonly shared among the different concentration of H_2_O_2_ and MSB treatments (Figure 2B). In addition, there were less than 200 common DEGs shared between H_2_O_2_ and MSB treatments, whereas 525 DEGs were commonly shared between 3 mM and 6 mM H_2_O_2_ and 378 DEGs were commonly shared between 0.28 mM and 0.42 mM MSB treatment. Moreover, oxidative stress enhanced the expression of genes involved in oxidoreduction, including CAT, GPX, and SOD, and transcription factors (Yap1 and Skn7). The critical roles of these genes involved in oxidoreduction and transcription factors in oxidative stress have been reported in many microorganisms, such as yeast, *Aspergillus fumigatus* and *Alternaria alternata* [36,37]. In this study, the up-regulated expression of genes involved in oxidoreduction and two transcription factors were observed in *A. oryzae* cells in response to H_2_O_2_ and MSB treatment. However, the expression levels were different. MSB treatment increased GPX, whereas exogenous H_2_O_2_ enhanced Yap1 transcription factor. Further, seven Skn7 transcription factors exhibited significant increase in the gene expressions when treated with H_2_O_2_ and the remaining five Skn7 transcription factors showed up-regulated expressions at MSB treatments. These results demonstrated the different response of *A. oryzae* cells to H_2_O_2_ and MSB treatment. Previous studies on oxidative stress response of filamentous fungi induced by H_2_O_2_ and paraquat also showed that cell responses were different with different treatment, in which paraquat increased mainly SOD, whereas exogenous H_2_O_2_ increased CAT [38]. Although the expression patterns of these genes involved in oxidoreduction and transcription factors were different, GSH content was found to be high in both treatments. GSH is one of the major antioxidant molecules in yeast cells, and is considered to play a vital role in cell defense mechanism against ROS [39]. Glutathione can react with oxidants non-enzymically, or the reaction can be catalyzed by glutathione peroxidase, and oxidized glutathiol (GSSG) could be recycled by glutathione reductase (GR) at the expense of NADPH [22]. Mutants of *S. cerevisiae* deficient in glutathione synthesis or glutathione recycling showed increased sensitivity to H_2_O_2_ [40]. In *A. niger*, GSH concentration increased significantly following the addition of H_2_O_2_ when compared to control [24]. These reports supported the results in this study that GSH has essential role in oxidative defense. Besides GSH, the composition of lipids in cells has close relationship with the response to oxidative stress. Fatty acids of yeast cells are more resistant to oxidative attack than those of mammalian cells due to the lack of enzymes capable of synthesizing polyunsaturated fatty acids (PUFAs) [41]. Cipak et al. observed that the PUFA-producing yeast was initially more sensitive to oxidative stress than the wild-type strain because induced PUFA production increased the levels of endogenous ROS [42]. However, the changes of lipids in *A. niger* were different from that observed in yeasts, indicating that H_2_O_2_ treatment significantly increased the unsaturation degree of fatty acids [43]. In this study, it was observed that the changes of fatty acid compositions in *A. oryzae* under oxidative stress were highly similar with yeast, which exhibited an increased content of saturated fatty acids.

## Figures and Tables

**Table 1:** Summary of the sequencing data and gene numbers of *A. oryzae* transcriptome under oxidative stress.

Samples	WT	3 mM H_2_O_2_	6 mM H_2_O_2_	0.28 mM MSB	0.42 mM MSB
Clean Reads	40,788,832	41,412,228	41,102,850	43,613,782	39,908,124
GC Content	52.28%	52.47%	52.26%	51.64%	51.88%
% ≥ Q30	94.27%	94.33%	94.40%	94.36%	94.35%
Mapped reads	36,552,991 (89.62%)	34,900,808 (84.28%)	34,852,216 (84.79%)	38,343,753 (87.92%)	35,212,300 (88.23%)
Unique mapped	36,452,891 (89.37%)	34,787,458 (84.00%)	34,722,583 (84.48%)	38,241,527 (87.68%)	35,122,814 (88.01%)
Multiple mapped	100,100 (0.25%)	113,350 (0.27%)	129,633 (0.32%)	102,226 (0.23%)	89,486 (0.22%)
